# Supplementary material for: Blind spots in global soil biodiversity and ecosystem function research
Source: Nat Commun. 2020 Aug 3;11:3870. doi: 10.1038/s41467-020-17688-2 (PMC7400591; doi:10.1038/s41467-020-17688-2)
Supplement: Supplementary file 1 — Supplementary Information [file 41467_2020_17688_MOESM1_ESM.pdf]

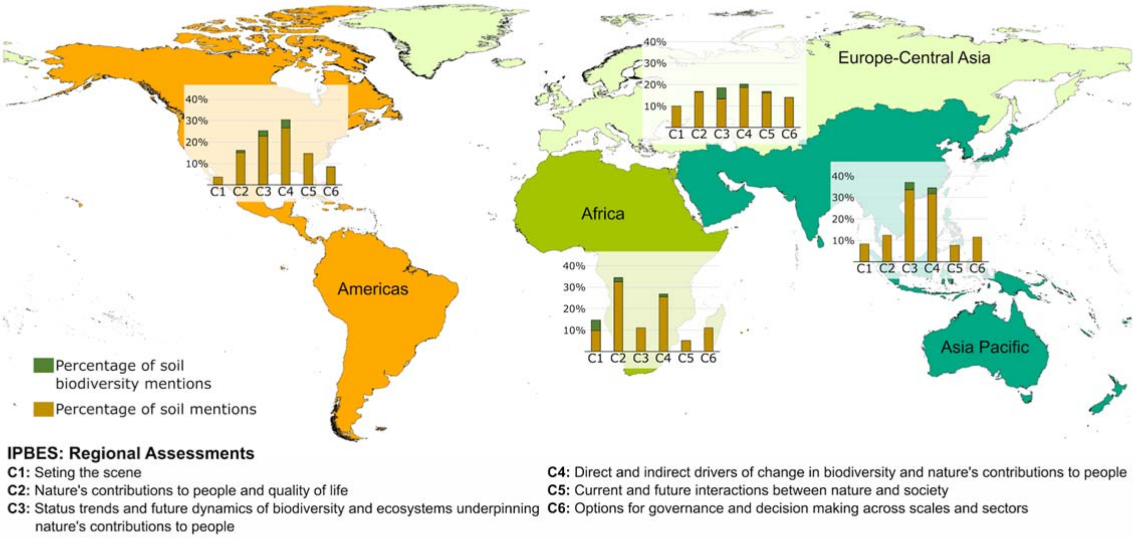

Supplementary Figure 1 Evaluation of the extent to which the recent Intergovernmental Science-Policy Platform on Biodiversity and Ecosystem Services Regional Assessments (i.e., Americas, Africa, Europe and Central Asia, and Asia Pacific) represent soil conditions (yellow bars) and especially soil biodiversity (green bars). The stacked bar plots show the proportional representation - regarding each chapter of the assessments - of pages with references to soils, soil conditions, functions and biodiversity, in relation to the total number of pages of each chapter. The green bars refer to the proportion of pages mentioning soil biodiversity. Overall, with very few exceptions (e.g., chapter 3 of the Europe and Central Asia Assessment), most of these mentions relate to soil conditions (i.e., physical and chemical) that favour or are related to aboveground diversity, and, apart from soil carbon fixation, almost no soil functions are mentioned across the assessments. Across the assessments, soil biodiversity is systematically mentioned to highlight data gaps rather than discuss their distribution, state, or past trends. Finally, it is also clear that both biodiversity and soil ecosystem functions are almost completely absent of both chapter 5 (current and future interactions between nature and society) and from chapter 6 (options for governance and decision making across scales and sectors), two of the most fundamental chapters for decision making and the development of future conservation policies.

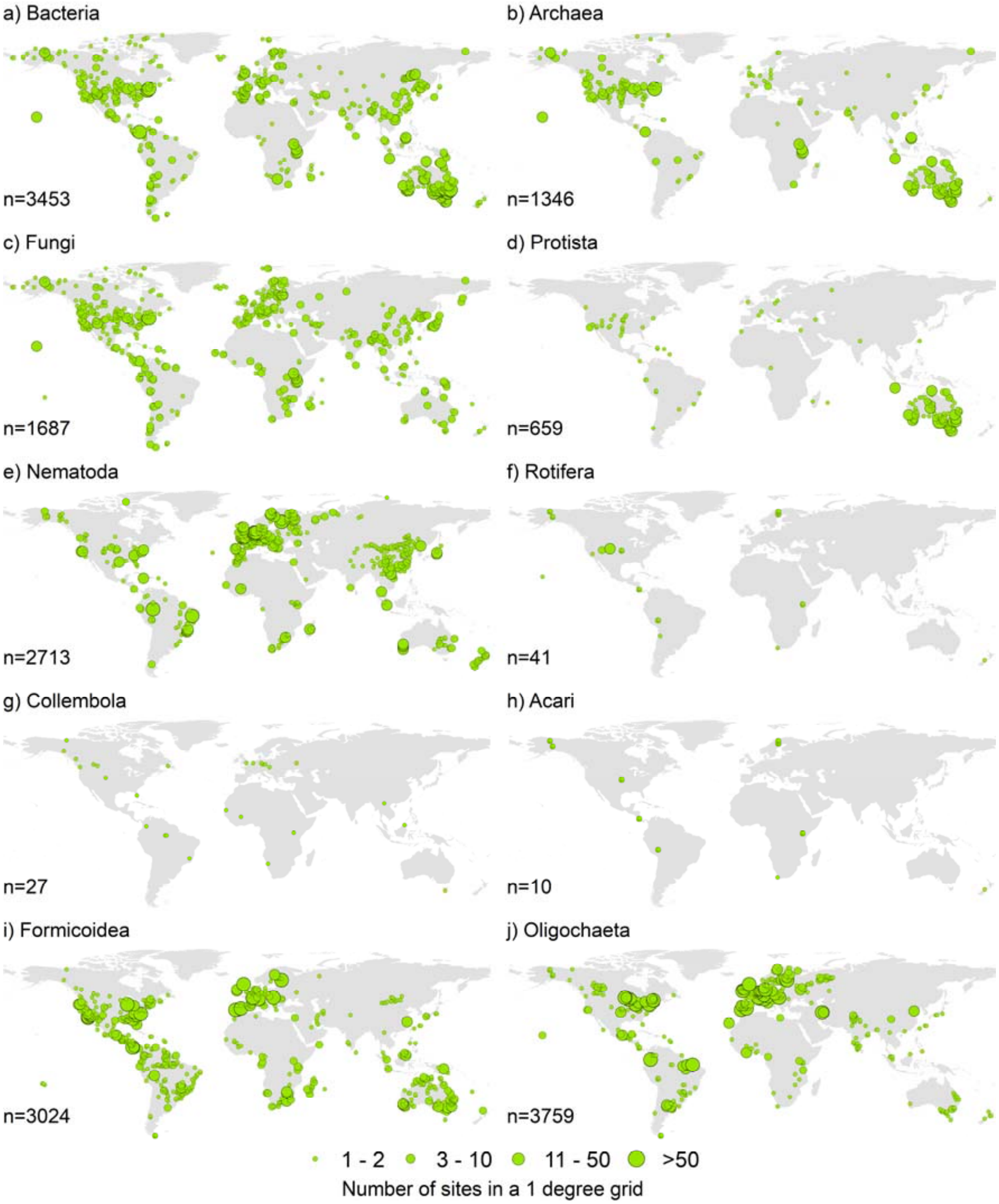

Supplementary Figure 2 Spatial distribution of the documented sampling sites across studies for different taxa: a) Bacteria; b) Archaea; c) fungi; d) Protista; e) Nematoda; f) Rotifera; g) Collembola; h) Acari; i) Formicoidea; and j) Oligochaeta.

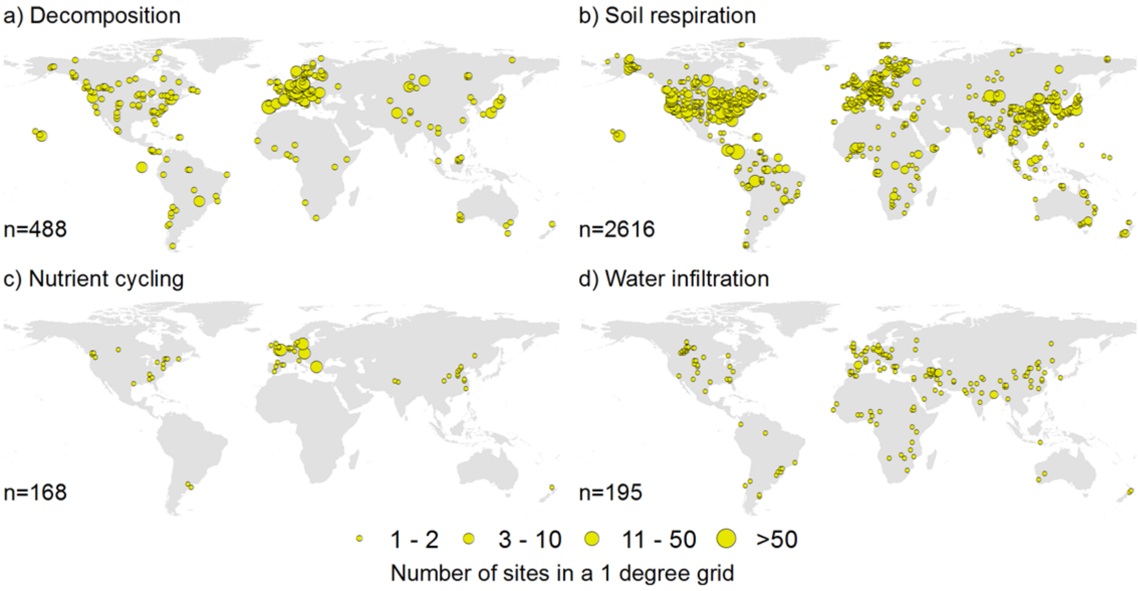

Supplementary Figure 3 Spatial distribution of the documented sampling sites across studies for different soil ecosystem functions: a) decomposition; b) soil respiration; c) nutrient cycling; and d) water infiltration.

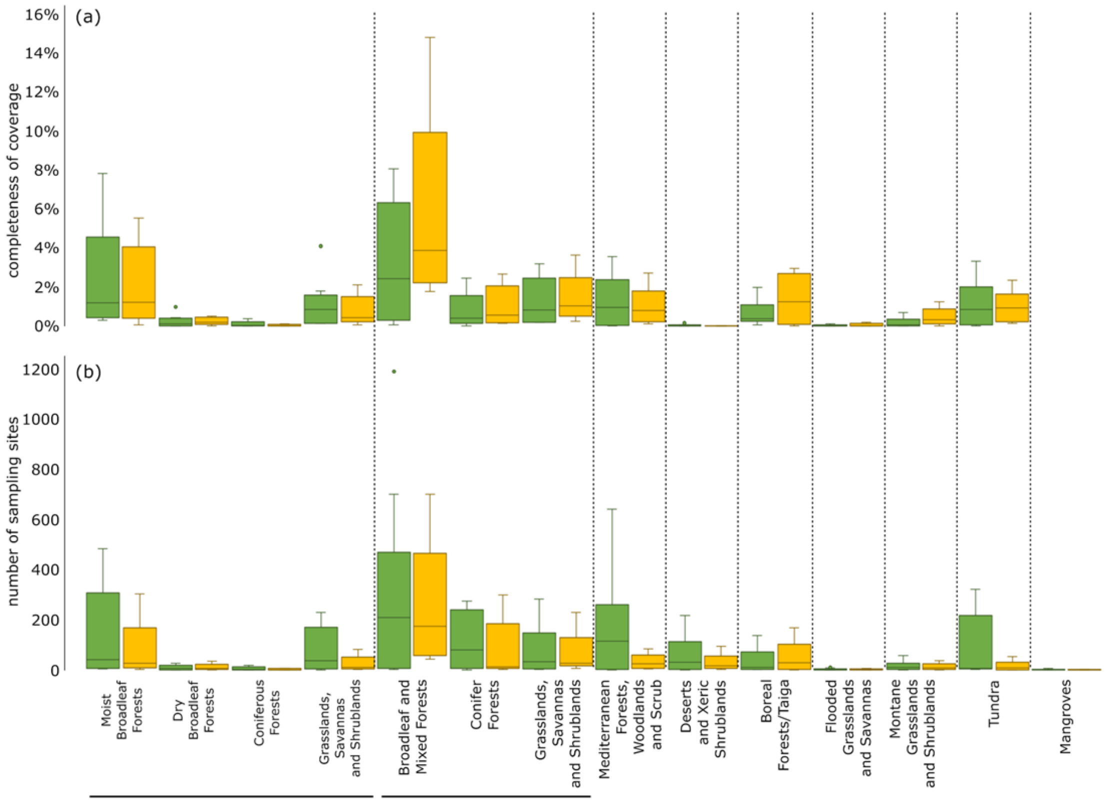

Supplementary Figure 4 Distribution of the documented sampling sites across the different terrestrial biomes. Box plots represent the quantile distribution across the different taxa (i.e., Bacteria, Archaea, fungi, Protista, Nematoda, Rotifera, Collembola, Acari, Formicoidea, and Oligochaeta; in green) and soil ecosystem functions (i.e., decomposition, soil respiration, nutrient cycling, water infiltration, and secondary productivity; in yellow): (a) level of inventory spatial completeness as the percentage of 1 degree cells covered by data across biomes (the coverage extent in Mangroves was not calculated); and (b) number of sampling sites.

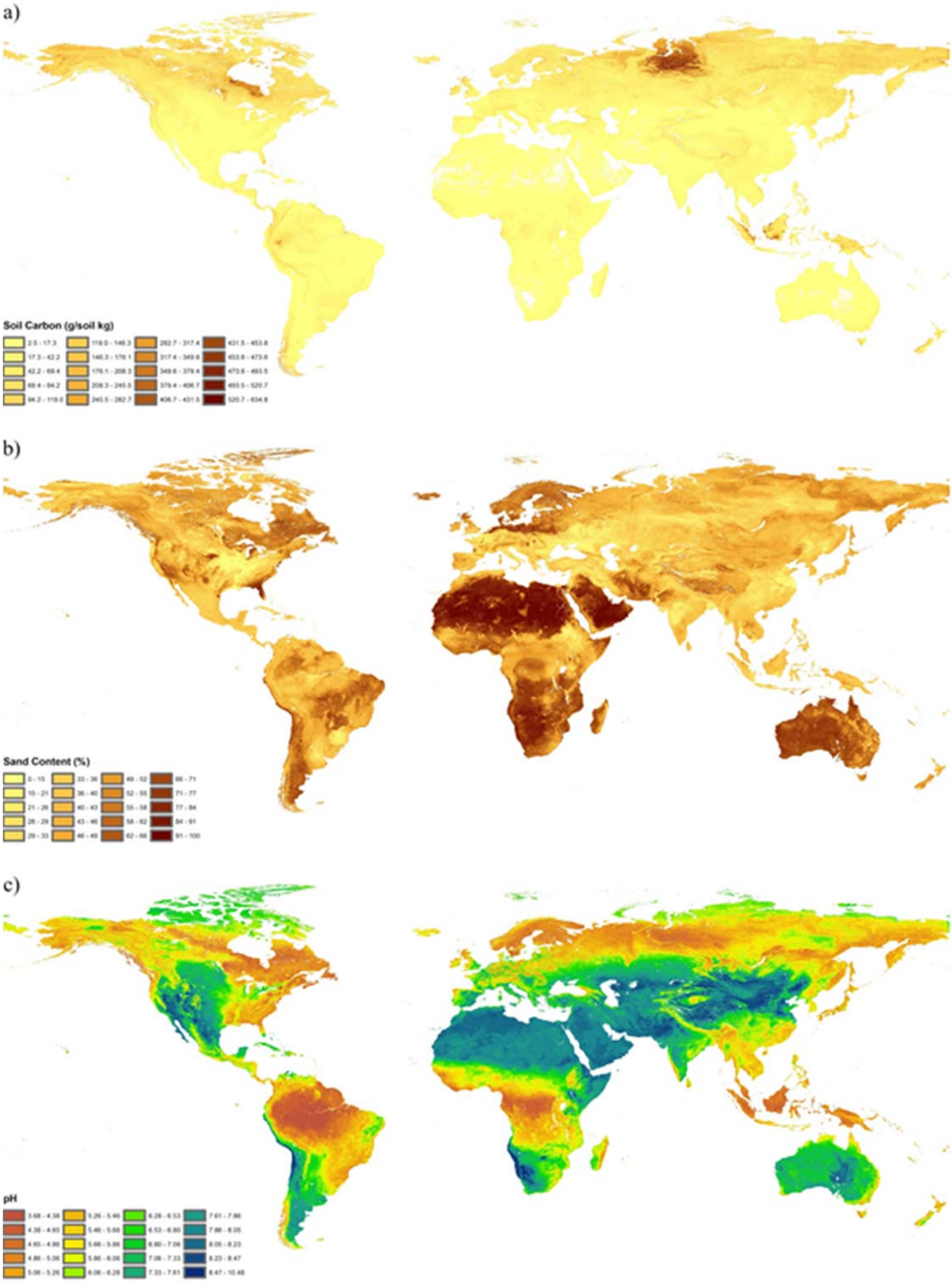

Supplementary Figure 5 Global distribution of the classified variables composing the soil realm: a) soil carbon[65]; b) sand content[65]; and c) pH[65].

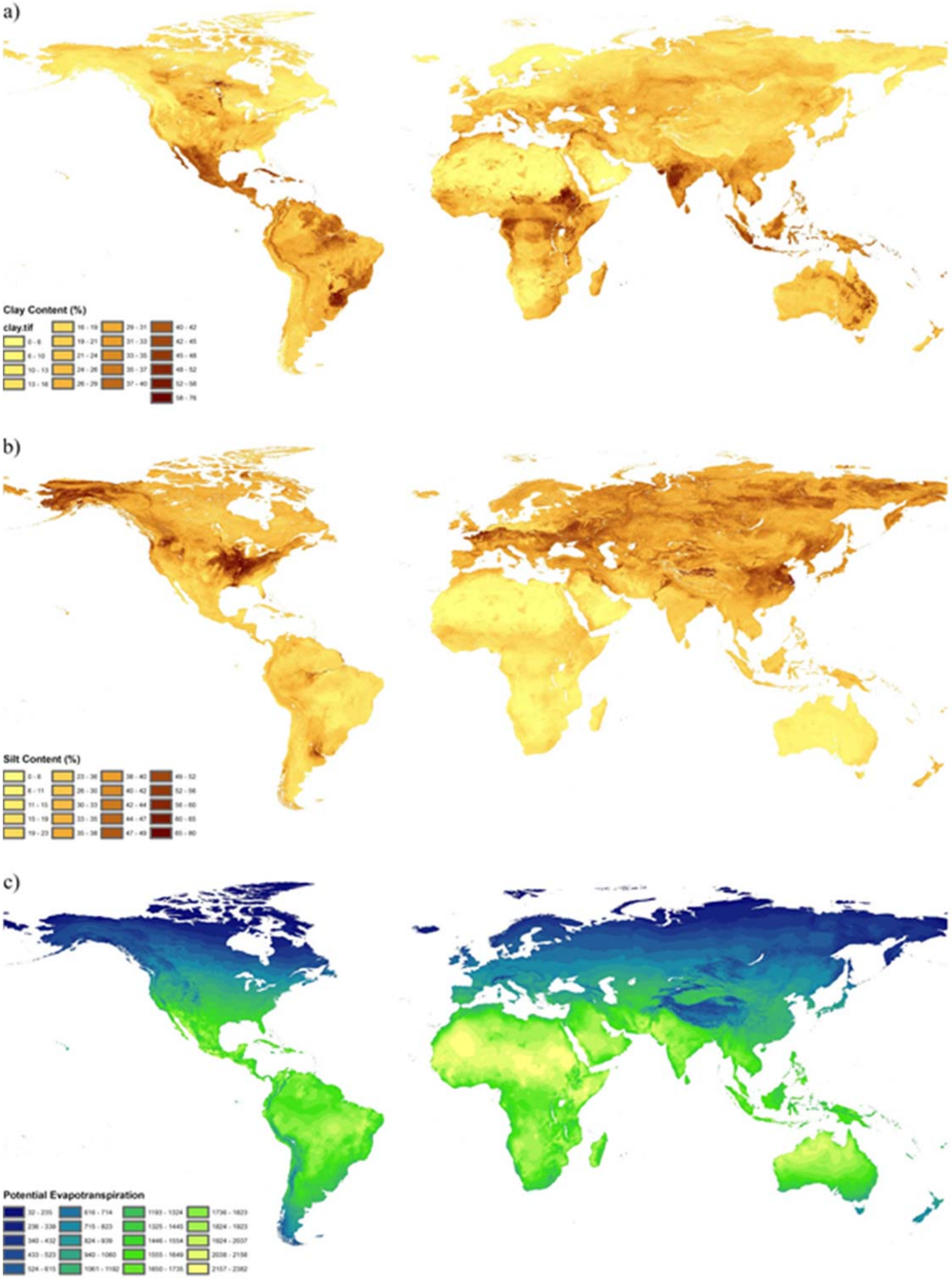

Supplementary Figure 6 Global distribution of the classified variables composing the soil realm: a) clay content[65]; b) silt content[65]; and c) potential evapotranspiration[167,168].

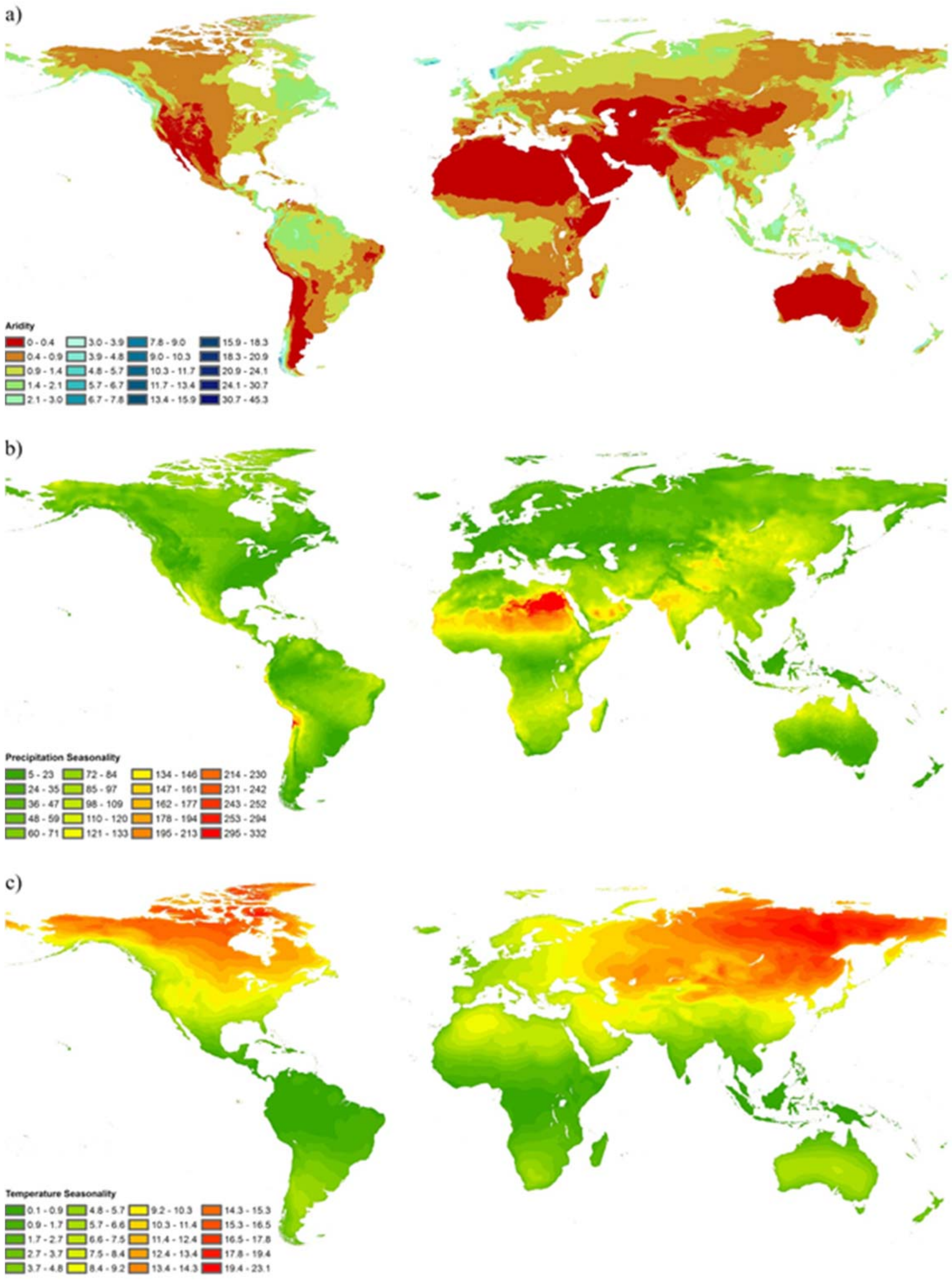

Supplementary Figure 7 Global distribution of the classified variables composing the soil realm: a) aridity[167,168]; b) precipitation seasonality[67]; and c) temperature seasonality[67].

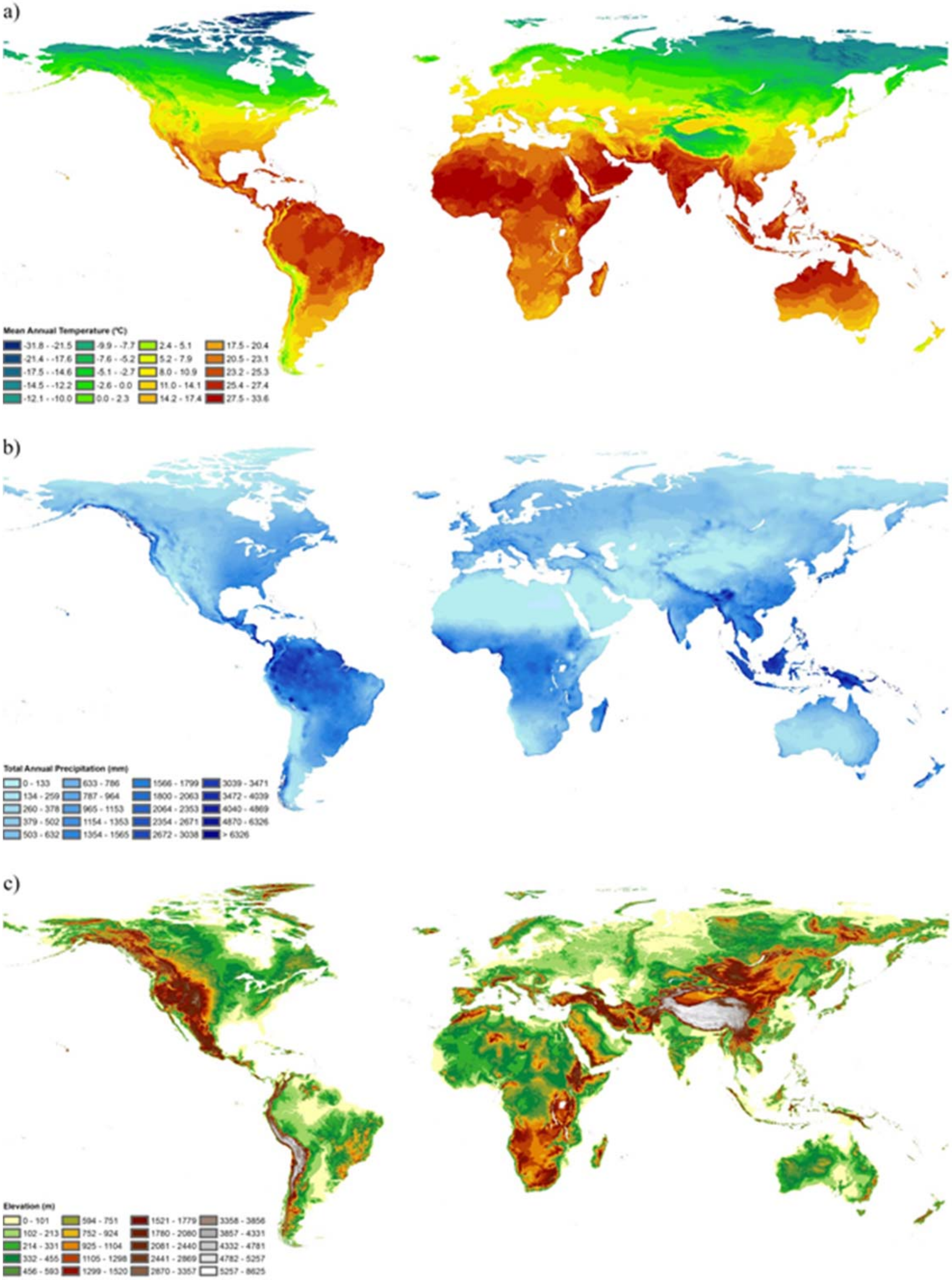

Supplementary Figure 8 Global distribution of the classified variables composing the soil realm: a) mean annual temperature[67]; b) total annual precipitation[67]; and c) elevation[68].

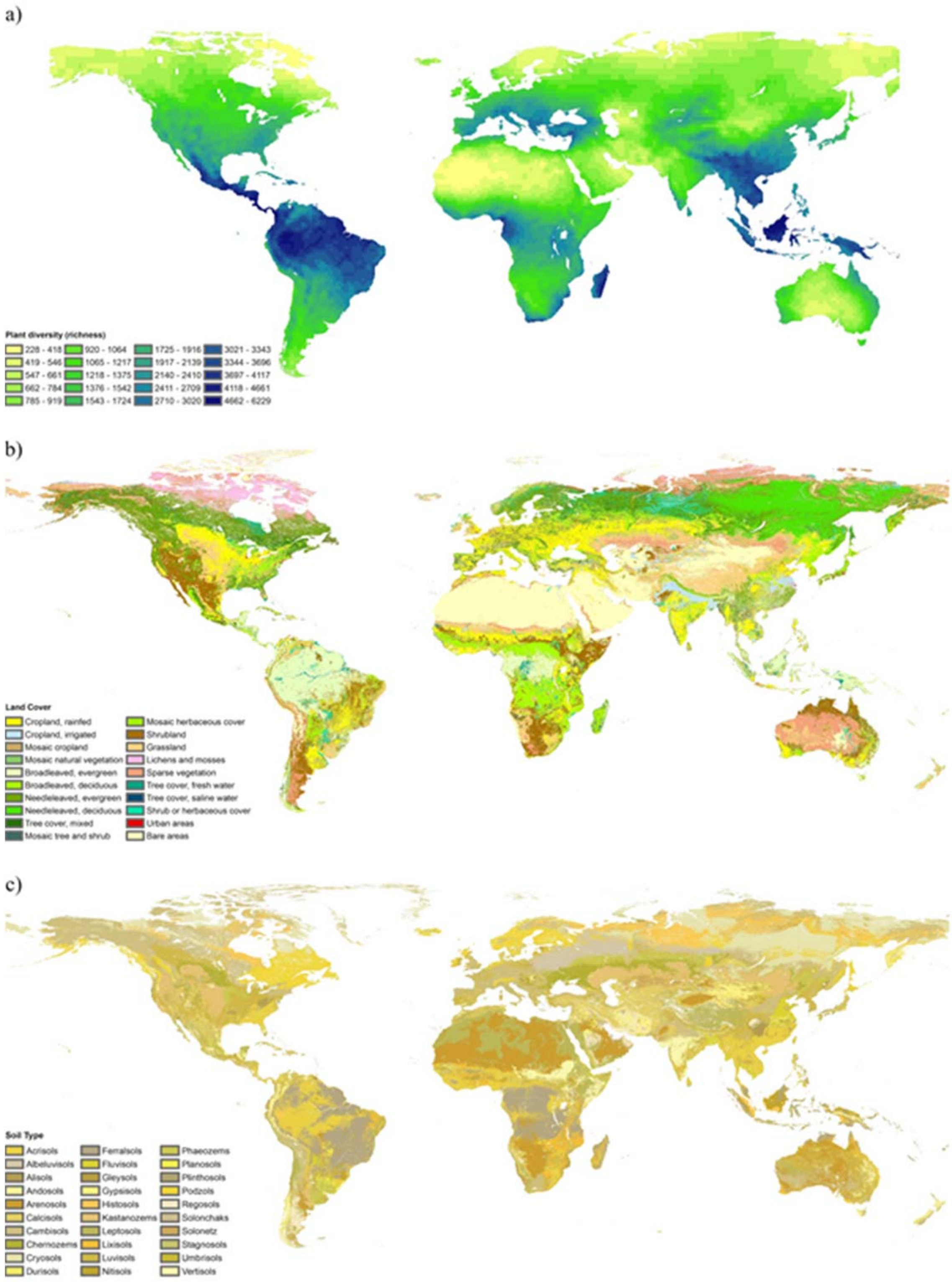

Supplementary Figure 9 Global distribution of the classified variables composing the soil realm: a) plant richness[69]; b) land cover[70]; and c) soil type[65].

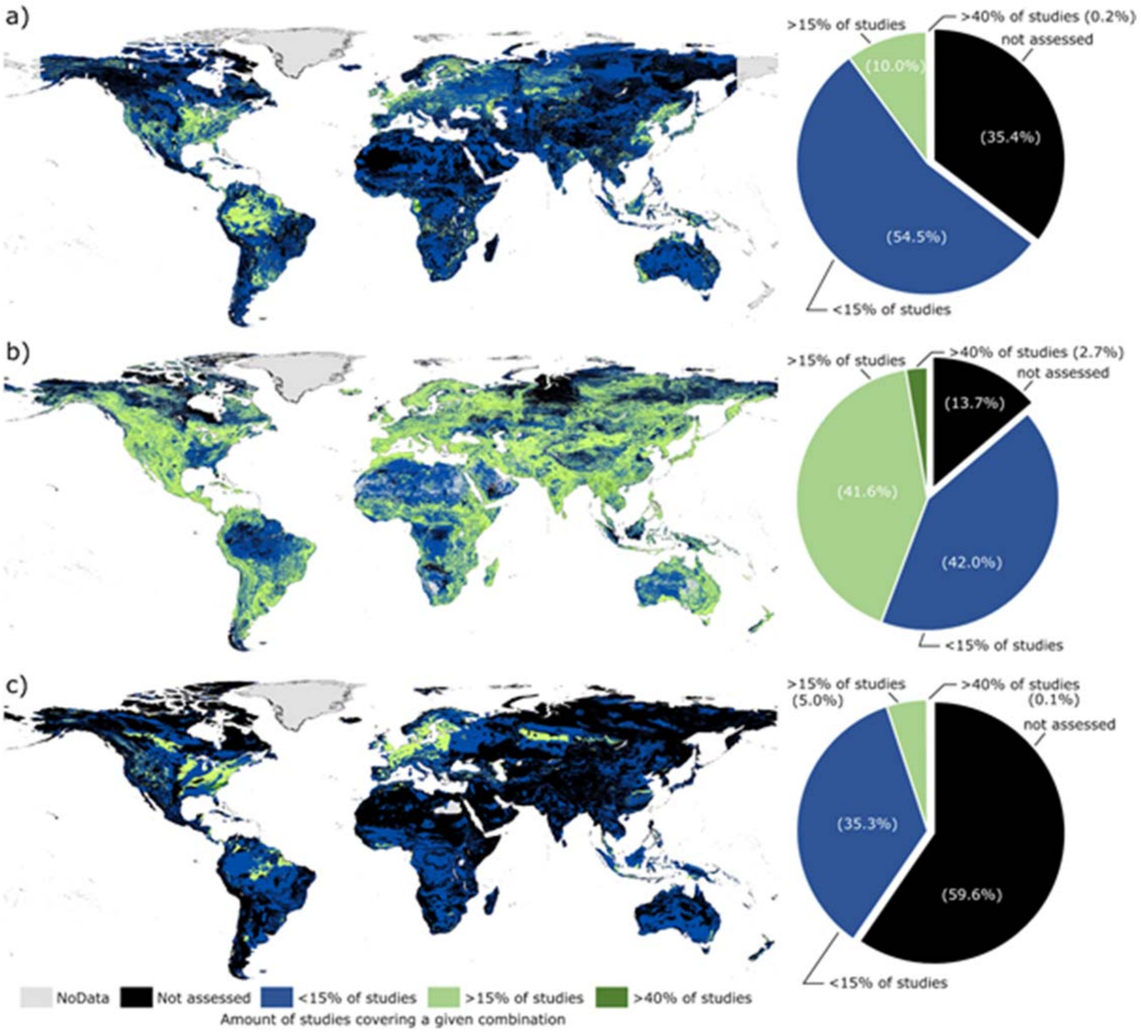

Supplementary Figure 10 The extent to which main soil environmental characteristics are assessed across macroecological studies. Colours correspond to the amount of studies covering a given combination of characteristics (see Methods for more details) within: a) land cover (including the combination of land cover, plant diversity and elevation); b) soils (including the combination of organic carbon content, sand content and pH); and c) climate (including the combination of mean precipitation and temperature, and their seasonality). Black corresponds to combinations that were not assessed by any of the studies here included; in blue are the combinations assessed by less than 15% of the studies (N= 7); in light green the variable combinations assessed by less than 40% of the studies (N=18); and in dark green, the variable combinations assessed by more than 40% of the studies. All combinations were created by a spatial overlap using the same class distribution of each variable as in Fig. 2 (see Methods and Supplementary Figures 5 to 9 for more details).

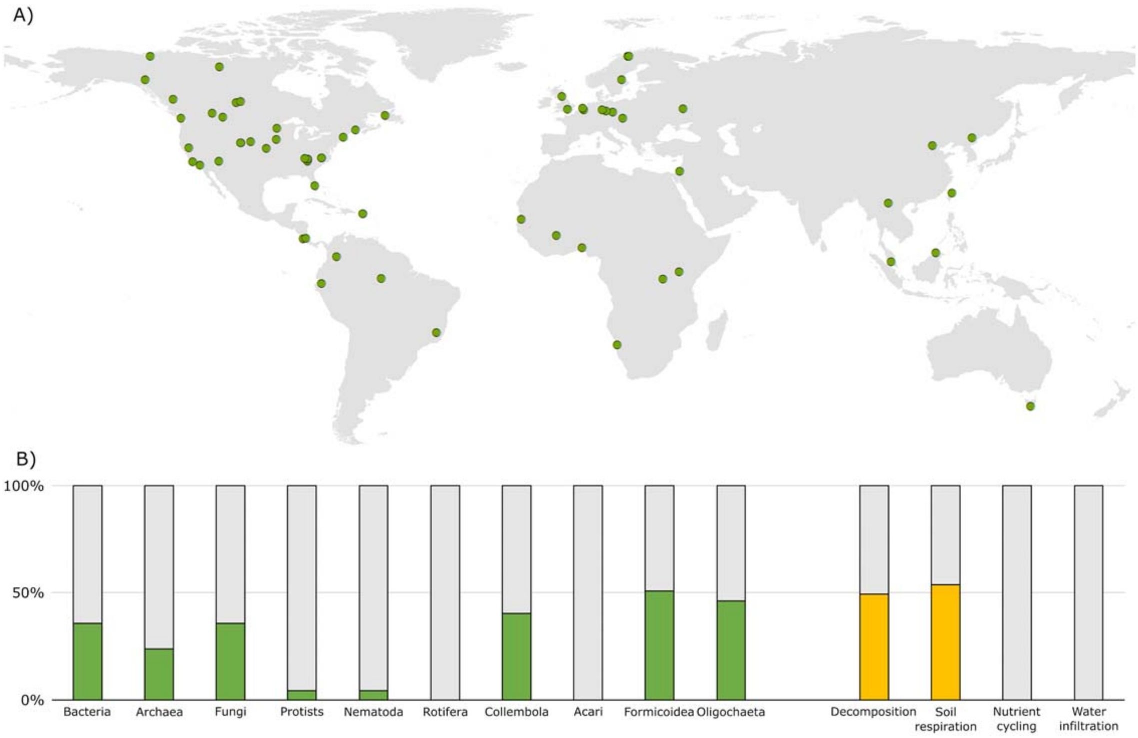

Supplementary Figure 11 Spatial distribution of the 67 locations with biodiversity and ecosystem function data. Overall spatial distribution (A) and the relative proportion of taxa and functions across all sites (B).

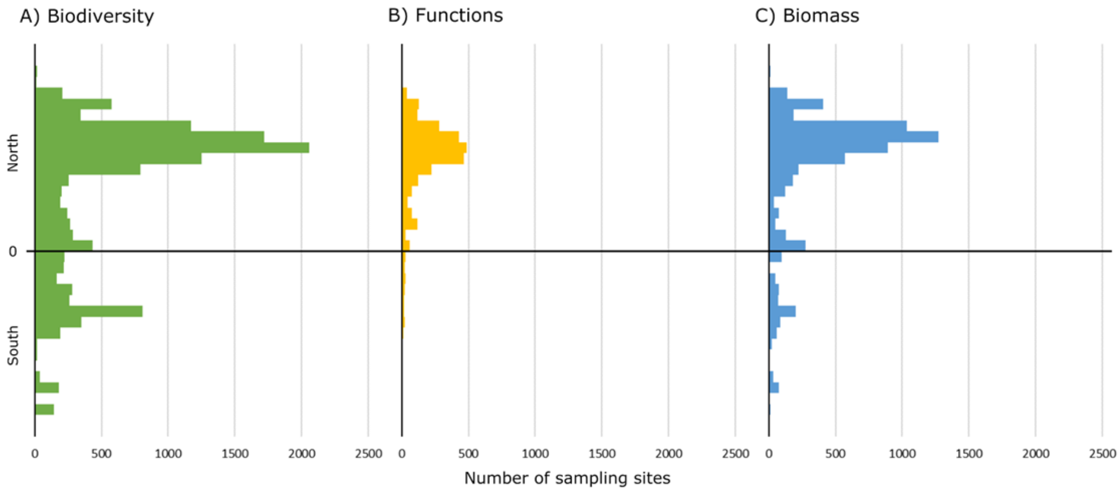

Supplementary Figure 12 Latitudinal distribution of sampling sites for A) biodiversity, B) ecosystem functions, and C) biomass.
