# Supplementary material for: Blind spots in global soil biodiversity and ecosystem function research
Source: Nat Commun. 2020 Aug 3;11:3870. doi: 10.1038/s41467-020-17688-2 (PMC7400591; doi:10.1038/s41467-020-17688-2)
Supplement: Supplementary file 3 — Reporting Summary [file 41467_2020_17688_MOESM3_ESM.pdf]

natureresearch

Corresponding author(s): CAG

Last updated by author(s): Mar 23, 2020

Reporting Summary

Nature Research wishes to improve the reproducibility of the work that we publish. This form provides structure for consistency and transparency in reporting. For further information on Nature Research policies, see Authors & Referees and the Editorial Policy Checklist .

Statistics

For all statistical analyses, confirm that the following items are present in the figure legend, table legend, main text, or Methods section.

n/a	Confirmed	
☐	☒	The exact sample size (n) for each experimental group/condition, given as a discrete number and unit of measurement
☐	☒	A statement on whether measurements were taken from distinct samples or whether the same sample was measured repeatedly
☐	☒	The statistical test(s) used AND whether they are one- or two-sided<br>*Only common tests should be described solely by name; describe more complex techniques in the Methods section.*
☒	☐	A description of all covariates tested
☐	☒	A description of any assumptions or corrections, such as tests of normality and adjustment for multiple comparisons
☐	☒	A full description of the statistical parameters including central tendency (e.g. means) or other basic estimates (e.g. regression coefficient) AND variation (e.g. standard deviation) or associated estimates of uncertainty (e.g. confidence intervals)
☒	☐	For null hypothesis testing, the test statistic (e.g. F, t, r) with confidence intervals, effect sizes, degrees of freedom and P value noted<br>*Give P values as exact values whenever suitable.*
☒	☐	For Bayesian analysis, information on the choice of priors and Markov chain Monte Carlo settings
☒	☐	For hierarchical and complex designs, identification of the appropriate level for tests and full reporting of outcomes
☒	☐	Estimates of effect sizes (e.g. Cohen's d, Pearson's r), indicating how they were calculated

Our web collection on statistics for biologists contains articles on many of the points above.

Software and code

Policy information about availability of computer code

Data collection	Data collection did not use any particular software apart from an Web of Knowledge search.
Data analysis	For data analysis and identification of blind spots, we used ArcGIS to prepare all environmental variables and ArcPy and Python to process them in relation to each point data source.

For manuscripts utilizing custom algorithms or software that are central to the research but not yet described in published literature, software must be made available to editors/reviewers. We strongly encourage code deposition in a community repository (e.g. GitHub). See the Nature Research guidelines for submitting code & software for further information.

Data

Policy information about availability of data

All manuscripts must include a data availability statement. This statement should provide the following information, where applicable:

- Accession codes, unique identifiers, or web links for publicly available datasets
- A list of figures that have associated raw data
- A description of any restrictions on data availability

During the review process, the data that support the findings of this study are available from the corresponding author upon reasonable request. After that, all data is going to be available as a single dataset including all soil biodiversity and function locations with reference to the respective manuscripts from which they were extracted. Also, all data in the figures will also be made available as a composite GeoTIF file. All datasets will be made available in a public repository upon publication. All underlying data regarding the environmental variables used is already made open source by the different data providers listed in the manuscript.

Field-specific reporting

Please select the one below that is the best fit for your research. If you are not sure, read the appropriate sections before making your selection.

☐ Life sciences ☐ Behavioural & social sciences ☒ Ecological, evolutionary & environmental sciences

For a reference copy of the document with all sections, see nature.com/documents/nr-reporting-summary-flat.pdf

Ecological, evolutionary & environmental sciences study design

All studies must disclose on these points even when the disclosure is negative.

Study description	we identify and characterize the existing gaps in soil biodiversity and ecosystem function data across soil macroecological studies and >11,000 sampling sites. These include significant spatial, environmental, taxonomic, and functional gaps, and an almost complete absence of temporally explicit data. We also identify the limitations of soil macroecological studies to explore general patterns in soil biodiversity-ecosystem functioning relationships. Based on this information, we provide clear priorities to support and expand soil macroecological research.
Research sample	From the literature search within the web of knowledge, 64 studies were selected and then classified according to the taxon and/or function. Given the constrains related to obtain standard location data across all studies, overall, ~72.6% (45 studies) of the total number of studies identified were included in the analysis. These cover ten different taxa and five (from a total of seven) soil ecosystem functions. We collected details on locations of 17,186 individual sampling sites representing studies on soil biodiversity, ecosystem function, with an overlap of 0.3%, and microbial biomass.
Sampling strategy	We created a dataset by collecting published literature on macroecological studies of soil biodiversity and ecosystem functions. For this literature search, we conducted a search in the Web of Science database in November 2018 within papers published between 1945 and 2018 using the keywords: (Global* OR Continental OR latitud*) AND (soil* OR belowground) AND (*function* OR *diversity OR organism* OR biota OR animal* OR invert* OR fauna*) AND distribution AND (*mycorrhiz* OR microb* OR nematod* OR bacteria* OR ant* OR fung* OR invertebrate* OR earthworm* OR termite* OR protist* OR eukaryot* OR collembola* OR rotifer* OR archaea OR formic* OR mite* OR arthropod* OR respiration OR decomposition OR nitrogen-cycling OR nutrient cycling OR water infiltration OR aggregate* OR bioturbation OR biomass). These keywords were selected to encompass the maximum number of published studies, which often use a variety of expressions for describing soil biodiversity and function. Additionally, we included studies found in the references of the papers returned by the database, as well as opportunistically gathering additional studies, such as from personal bibliographic databases of global soil studies.
Data collection	For many of the studies included in this analysis, the underlying data on the locations used in the studies was already available on-line in data repositories and was extracted without further analysis. For the other studies where data was not already available on-line we contacted the main authors with a request for a dataset with the sampling locations from each study. We took the data as it was published or shared by the authors of the studies and we did not attempt to do any data correction to the locations included in each dataset.
Timing and spatial scale	The original search returned studies ranging from 1995 to 2018. The studies included ranged from 2004 to 2019. All studies have a global scale as defined in the manuscript.
Data exclusions	From the original search studies were included based on a predefined set of criteria: (1) studies dealing with soil taxa and/or soil ecosystem functions; (2) studies spanning more than one continent (the Pacific islands not included in any particular continent were counted as an individual continent, contributing to the inclusion of some studies); and (3) studies that span across an entire continent (i.e., a study focussing only on Europe would not be included but if larger scales would be assessed - e.g., across Eurasia - the study would be included). All other were excluded from the analysis.
Reproducibility	Within the manuscript, we clearly state all the steps taken to ensure the reproducibility of the study including following to the extent that was possible for this type of study the Preferred Reporting Items for Systematic Reviews and Meta-Analyses (PRISMA) protocol.
Randomization	Not applicable.
Blinding	Not applicable.

Did the study involve field work? ☐ Yes ☒ No

Reporting for specific materials, systems and methods

We require information from authors about some types of materials, experimental systems and methods used in many studies. Here, indicate whether each material, system or method listed is relevant to your study. If you are not sure if a list item applies to your research, read the appropriate section before selecting a response.

Materials & experimental systems

n/a	Involved in the study
☒	☐ Antibodies
☒	☐ Eukaryotic cell lines
☒	☐ Palaeontology
☒	☐ Animals and other organisms
☒	☐ Human research participants
☒	☐ Clinical data

Methods

n/a	Involved in the study
☒	☐ ChIP-seq
☒	☐ Flow cytometry
☒	☐ MRI-based neuroimaging
